# Therapeutic targets and new directions for antibodies developed for ovarian cancer

**DOI:** 10.1080/19420862.2016.1219005

**Published:** 2016-08-05

**Authors:** Heather J. Bax, Debra H. Josephs, Giulia Pellizzari, James F. Spicer, Ana Montes, Sophia N. Karagiannis

**Affiliations:** a St. John's Institute of Dermatology, Division of Genetics and Molecular Medicine, Faculty of Life Sciences and Medicine, King's College London & NIHR Biomedical Research Center at Guy's and St. Thomas' Hospital and King's College London, Guy's Hospital, King's College London, London, UK; b Division of Cancer Studies, Faculty of Life Sciences and Medicine, King's College London, Guy's Hospital, London, UK; c Department of Medical Oncology, Guy's and St Thomas' NHS Foundation Trust, London, UK

**Keywords:** Antibodies, clinical trials, immunotherapy, immune checkpoint, molecular-targeting, ovarian cancer, tumor-associated antigen, tumor-promoting molecule, vaccine

## Abstract

Antibody therapeutics against different target antigens are widely used in the treatment of different malignancies including ovarian carcinomas, but this disease still requires more effective agents. Improved understanding of the biological features, signaling pathways, and immunological escape mechanisms involved in ovarian cancer has emerged in the past few years. These advances, including an appreciation of the cross-talk between cancer cells and the patient's immune system, have led to the identification of new targets. In turn, potential antibody treatments with various mechanisms of action, including immune activation or toxin-delivery, that are directed at these targets have been developed. Here, we identify established as well as novel targets for antibodies in ovarian cancer, and discuss how they may provide fresh opportunities to identify interventions with enhanced therapeutic potential.

## Introduction

It is estimated that ∼22,280 American women will receive new diagnoses of ovarian cancer in 2016, and an estimated 14,240 deaths from the disease will occur in this year.^1^ Standard therapy is a combination of maximal surgical cytoreductive and taxane- and platinum-based chemotherapeutic agents. Nevertheless, relapse is common after first-line treatment. Despite investigations of novel chemotherapeutic regimes, targeted and other therapies, there have been no significant improvements in clinical outcomes or cure rates, with current 5-year overall survival rates at only 45%.^2^

Given that ovarian cancer is known to be immunogenic and high numbers of infiltrating immune cells, including effector cells such as T cells and macrophages, are associated with improved survival rates,^3^ antibody-based therapies are thought to offer promise. Monoclonal antibody immunotherapies may redirect these effector cells against cancer and mediate specific and potent anti-tumor immune responses, with the aim of restricting tumor growth and improving disease course. Here, we focus on established and emerging new targets for antibody treatments in ovarian carcinoma, and we discuss monoclonal antibodies that have been studied in patients with this disease.

## Targets for antibody treatments

### Tumor-associated antigens

Tumor-associated antigens (TAAs) are surface-associated molecules or receptors expressed by tumor cells, which have limited or no expression on normal cells. Often TAAs are involved in the activation of signaling transduction pathways that support unregulated growth or division of cancer cells. This specificity in expression and role in pro-tumoral functions make TAAs promising antibody targets, allowing tumor cells to be specifically marked for immune cell destruction or blockade of tumor-associated signaling, which impedes malignant function, invasiveness and survival.

#### CA125

CA125 (MUC16), an extremely large, 2500–5000 kDa, mucin-like surface glycoprotein, is expressed in greater than 95% of non-mucinous stage III/IV epithelial ovarian cancers (EOCs) and in 50–80% of ovarian tumors overall. CA125 is thought to support tumor-associated immune escape in the tumor microenvironment (TME).^4,5^ High CA125 expression correlates with protection against cytolytic killing by natural killer (NK) cells, which is linked to reduced activating immune synapses between NK and target cells, and thus decreased cell adhesion.^5^ This may be because the NK synaptic cleft requires a distance of 10–50 nm between NK and cancer cells, which is thought to be disrupted by the large (up to 24,000 amino acid) protein backbone of CA125 that can protrude from ovarian tumor cells by up to 1–5μm. ^5^ However, given the heterogeneity of the size of CA125 reported, which may be a result of the biological source of the molecules studied or differing biological methods used to characterize them,^6^ or significant variation in the extent of protein glycosylation,^5^ the degree of immune escape as well as other biological functions of CA125-expressing tumor cells may vary.

It has also been suggested that inhibition may be due to a CA125-induced reduction in NK cell expression of the Fc activating receptor, CD16.^4^ In fact, NK cells from patients with EOCs have shown significant reduction in CD16 expression compared to NK cells from healthy donors. A down-regulation of activatory receptors, such as CD16, leads to relative predominance of NK cell inhibitory receptors, and thus NK cells fail to respond to tumor cells, allowing tumor evasion of the innate immune response.^4^ Similarly, a downregulation of CD16 may also lead to ovarian tumor cell evasion of the adaptive immune system, by prevention of CD16 binding to host tumor-specific immunoglobulins.^4^ These immunoediting mechanisms are likely to potentiate the progression and proliferation of ovarian tumors.

CA125 is also thought to facilitate pro-tumor cell-cell interactions in an N-linked glycan dependent manner.^7^ CA125 on the surface of ovarian tumor cells binds to the glycoprotein mesothelin, expressed on epithelial cells (described below), with a high K_d_ of 5–10 nM. This cell adhesion is likely to occur in the peritoneum of patients with ovarian cancer, and may provide the first step to metastasis of tumor cells, likely reinforced by recruitment of CD44, β-1 integrins and other adhesion molecules.^7^

CA125 is shed from ovarian cancer cells into the blood and peritoneal cavity upon proteolytic cleavage. CA125 serum levels are known to correlate with tumor progression and recurrence. Thus, monitoring serum CA125 levels is a well-established and useful surrogate for evaluating response to conventional chemotherapeutic and surgical treatments, and is routinely used for surveillance in follow-up.^8^

#### MUC1

MUC1 is an epithelial mucin, comprising a heavily glycosylated transmembrane glycoprotein, overexpressed in many carcinomas, including 90% of EOC cells.^9^ The subunit, MUC1-C, is thought to contribute to malignant cellular transformation by regulating gene transcription, blocking stress-induced apoptosis and necrosis, and attenuating death receptor activation. The extracellular domain consists of mucin-like tandem repeats, which are differentially glycosylated between malignant and normal cells, resulting in the exposure of differing peptide epitopes. MUC1 expression in cancer facilitates tumor invasive growth and metastasis. It can act in both an anti-adhesion manner, resulting in the release of cells from tumor nests and increased metastasis, and a pro-adhesion manner, leading to malignant spread.^9,10^ Cancer cells also exploit MUC1 interactions with a number of growth receptors to promote survival. High expression of MUC1 by tumor cells leads to reduced cytotoxic killing by T and NK cells. This may be due to the large and complex nature of this TAA, which results in masking of extra-cellular domains for immunosurveillance and mediates tumor cell escape.^9^

Humoral and cellular responses against MUC1 have been observed in cancer patients.^11^ Anti-MUC1 antibodies have been shown to inversely correlate with ovarian carcinoma risk factors,^12^ thus supporting MUC1 as a promising target for therapy.

#### EpCAM

EpCAM (CD326) is a type I transmembrane glycoprotein that is highly expressed across all ovarian cancer subtypes. Its expression is maintained in metastatic disease,^13^ and EpCAM overexpression is prognostic of reduced overall survival (OS) in ovarian cancer.^14^ Expression on epithelial carcinomas is at the cell surface, whereas in normal epithelial tissues, EpCAM is expressed basolaterally and is protected by tight junctions, making this an attractive target for anti-cancer therapy.

However, EpCAM is reported to have mixed functions in tumor formation, motility and metastasis. EpCAM-expressing cells make homophilic interactions, forming aggregated clusters, which may be important in the structure of a tumor, but may also impede invasion and metastasis. In contrast, EpCAM inhibits E-cadherin mediated cell-cell interactions, and therefore, in this manner, may promote cell motility and metastasis.^15^ It is likely that these contrasting functions may be induced differentially depending on the TME, and there is little evidence to ascertain how these dynamics affect ovarian cancer.

#### Folate receptor-α

Folate receptor-α (FRα) is a 38 kDa glycosylphosphatidylinositol (GPI)-anchored membrane protein, which can transport folate via receptor-mediated endocytosis. It is overexpressed in 80–90% of EOCs.^16^ The level of FRα expression on cancer cells, has been shown to correlate with the histologic grade and stage of ovarian cancer.^17-19^ Overexpression is significantly higher in serous ovarian carcinoma than in mucinous carcinomas and other epithelial histotypes. FRα expression is thus considered a marker of tumor aggressiveness. Although there is conflicting data reported in the literature, when all histotypes of ovarian cancer are studied^18^ elevated tumor expression of FRα is associated with lower disease-free interval (DFI) and poor OS in patients with serous ovarian carcinoma.^19^

A role for FRα in tumor cell survival and progression is demonstrated by the preservation of FRα expression on recurrent tumors and metastatic foci.^18^ It is thought that over-expression of FRα by cancer cells may support tumor progression by modulating folate uptake and regulatory signals, resulting in increased proliferation of malignant cells through enhanced DNA synthesis.^20^ Knockdown of FRα expression on the SKOV-3 ovarian carcinoma cells resulted in inhibition of folate-mediated cell proliferation, migration and invasion.^17^ FRα may also facilitate chemo-resistance in ovarian carcinoma.^19-21^ Higher tissue FRα expression correlates with poor chemotherapeutic response in patients with serous ovarian carcinoma. Lower levels of apoptosis in vitro were induced by cisplatin, paclitaxel or topotecan treatment of OVACR-3 cells expressing high levels of FRα, compared to low-expressing control cells. The mechanism of this apoptotic escape is thought to be via FRα-mediated down-regulation of caspase 3 and 7 apoptosis pathways, increased expression of the anti-apoptotic molecule, Bcl-2 and decreased expression of the pro-apoptotic molecule, Bax.^19^ Furthermore, inhibition of the tumor suppressor gene, caveolin-1, by FRα is thought to support ovarian carcinoma progression.^20^ In vitro studies using IGROV1 and SKOV3 ovarian cancer cells demonstrated that FRα expression levels correlate with the downregulation of caveolin-1 expression, allowing tumor-progression by promoting cell cycle progression and anchorage-independent growth; functions which are normally inhibited by caveolin-1.^22^

FRα is considered to be a promising target for anti-cancer therapies^23^, since it is highly expressed on carcinoma cells, but has limited expression on normal tissues; expression is found only at low levels on the retina, placenta, intestine and choroid plexus, at the luminal surface of the kidney and lung, which are not accessible to the bloodstream.^24^ Furthermore, it has been reported that patients with ovarian cancer demonstrate increased immunity to FRα compared to healthy controls, suggesting that this is a sensible target for immunotherapies.^25^

#### Mesothelin

Mesothelin is a 40 kDa immunogenic cell surface protein that is highly expressed, particularly in non-mucinous subtypes of ovarian cancer cells, with normal tissue expression restricted to mesothelial cells.^26^ As described above, an interaction between CA125 on tumor cells and mesothelin in the peritoneum may be involved in the metastatic process. Furthermore, co-expression of mesothelin and CA125 on tumor cells is thought to lead to clustering of tumor cells at metastatic sites, as CA125-expressing cells have demonstrated significantly higher homotypic adhesion interactions compared to CA125-negative tumor cells.^7^ Such multicellular spheroids have been observed in the ascitic fluid of patients with ovarian cancer, and these have been shown to adhere to human mesothelial cell layers. This suggests a role for CA125-mesothelin mediated cell clusters in peritoneal dissemination of disease.^27^ Although adhesion is partly reduced by β-1 integrin inhibition, molecules such as mesothelin are also likely to be important.^7^

Consistent with its proposed role in tumor metastasis, high expression of mesothelin in ovarian cancer has been associated with more advanced disease, and worse progression-free survival (PFS) and OS.^28^ Additionally, high levels of mesothelin expression correlate with resistance to chemotherapy.^28^

#### Epidermal growth factor receptor family

The epidermal growth factor receptor (EGFR) family comprises 4 structurally-related tyrosine kinase receptors expressed on the apical surface of epithelial cells; ErbB1/HER1 (commonly known as EGFR or HER1), ErbB2/HER2, ErbB3/HER3, and ErbB4/HER4. Upon ligand binding, receptor dimerization occurs, followed by tyrosine auto-phosphorylation and subsequent activation of EGFR signaling. Downstream signaling is known to augment cellular responses, including cancer cell proliferation, motility, invasion, and survival.^29^

HER1 is the most widely studied EGFR family member involved in ovarian cancer. Increased HER1 expression is an early event in ovarian cancer development, and thus may be involved in neoplasia.^29^ HER1 amplification analysis in tissue microarrays showed that HER1, but not HER2, expression was greater in higher grade ovarian carcinomas.^30^ EGFR pathway activation is also involved in tumor growth and survival. Stimulation by HER1 ligands has been demonstrated in vitro by inhibition of ovarian tumor cell growth upon HER1 activation blockade^31^ and in vivo by the ablation of ovarian tumor cell xenograft growth when mice are depleted of EGF.^32^ HER1 may support cancer cell invasion and migration through the regulation of cell adhesion proteins.^33,34^ When HER1 expression was decreased in OVCAR-8 ovarian cancer cells, a selective decrease in cell clustering and adhesion to laminin-1 was observed. Cells with inhibited HER1 expression also lost MMP-9 activity and had impaired migratory capacity.^33^ Depleted HER1 expression resulted in decreased E-cadherin expression by tumor cells.^34^ Furthermore, overexpression of HER1 by tumor cells is associated with resistance to hormone therapies, chemotherapy and radiotherapy.^29,35-37^ One proposed mechanism for this tumor cell escape is that HER1 activation leads to the up-regulation of the apoptosis inhibitor protein, survivin, which is found at high levels in many tumor types.^35^

Since members of the EGFR family are overexpressed on many solid tumors, including ovarian carcinomas, and expression is associated with poorer patient outcomes,^37^ they present promising targets for therapy. Specifically, HER1 is expressed in 25–50% of ovarian cancers^21^ and HER2 expression is thought to range from 5 to 66%, but expression levels are low in peripheral normal tissues.

#### PDGFRα

Platelet-derived growth factor receptor-α (PDGFRα) is a 170 kDa member of the class III receptor tyrosine kinase family. Upon binding of platelet-derived growth factor (PDGF), receptor dimerization occurs, leading to downstream events including stimulation of tumor cell growth and inhibition of apoptosis, and regulation of stroma and angiogenesis.^38^

Elevated PDGF and PDGFRα have been detected in ovarian cancer tissue across serous, mucinous and endometrioid histologies and different stage of disease.^38-40^ In ovarian cancer cell cultures, an autocrine mechanism of PDGF/PDGFRα-mediated tumor cell growth was identified.^41^ Furthermore, associations between prognosis and tumor cell PDGFRα expression have also been observed.^39,40^

#### NaPi2b

NaPi2b (*SLC34A2*) is a multi-transmembrane, sodium-dependent phosphate transporter (also termed the TAA, MX35). This TAA is expressed in ∼90% of ovarian cancers; overexpression of NaPi2b was detected in specimens from all histological types of EOC, but not mucinous tumors. Expression is restricted in normal tissues, making NaPi2b a potential target of immunotherapeutic and diagnostic approaches for EOC. In addition, NaPi2b levels tend to be higher in well-differentiated EOC, suggesting that expression may be a marker of low grade tumors and better prognosis.^42^

#### EFNA4

Ephrin-A4 (EFNA4), a member of the Ephrin (Eph) family of receptors, is a tumor antigen that is overexpressed by a number of different tumors, including primarily triple negative breast cancers and ovarian cancers. Recently EFNA4 has also been identified as a novel tumor-initiating cell (TIC)-associated target.^43^ TICs are a subpopulation of tumor cells that drive tumor growth, resistance to treatment, and disease recurrence. These characteristics could render Ephrin-A4 a promising target for antibody therapies.

### Tumor-promoting molecules

**Figure 1.**
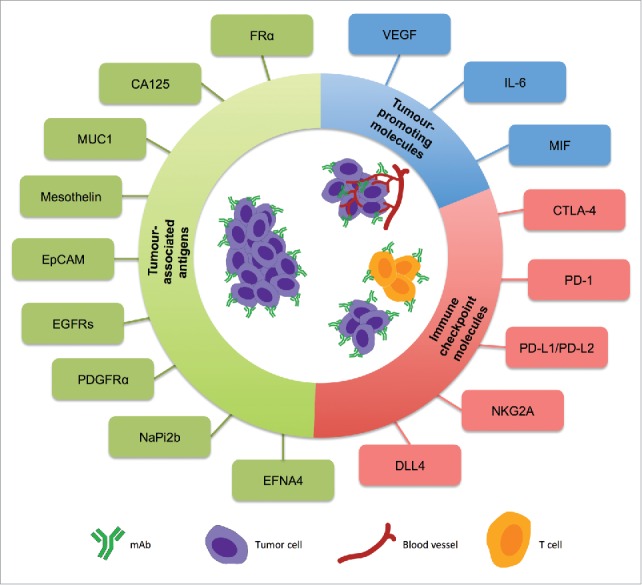
Schematic representation of the monoclonal antibody targets for ovarian cancer. Antibodies for treatment of ovarian cancer can be targeted against tumor-associated antigens, tumor-promoting molecules produced and secreted in tumor environments, or against immune checkpoint molecules to engender immune cell activation and overcome immune tolerance supported by tumors.

Tumor promoting molecules are secreted by cancer cells in order to support tumor growth and suppress or subvert anti-tumoral immune responses (Fig. 1). These have also become targets for antibody therapeutic strategies. Two key examples are discussed here.

#### VEGF

Vascular endothelial growth factor (VEGF) is a pro-angiogenic cytokine that binds specific tyrosine kinase receptors (VEGFRs). This leads to the activation of downstream pathways that enhance endothelial cell proliferation and survival, increase migration and invasion of endothelial cells, increase permeability of existing vessels, and enhance homing of vascular precursor cells from the bone marrow. All of these aid the formation of new blood vessels; a critical part of the survival, proliferation, invasion and metastasis of cancer cells.^44^ VEGF can also have autocrine effects on tumor cells, directly influencing their survival, migration and invasion, and immune suppression.^44^ VEGF and VEGFR, both expressed in EOC, form an autocrine loop that protects EOC cells from apoptosis in anchorage free growth conditions (anoikis). In vitro studies, using a number of ovarian carcinoma cell lines, have demonstrated that blockade of VEGF-VEGFR engagement results in an abolishment of receptor auto-phosphorylation and subsequent increases in apoptosis of single-cell tumor cultures.^45^ These conditions reflect those of tumor cells in ascitic fluid, implying a role for VEGF in ascites development.

Furthermore, secretion of VEGF by tumor cells is thought to create an immunosuppressive microenvironment, where maturation of dendritic cells is ablated, and T cell responses are inhibited.^46^ VEGF may also influence homing of bone marrow-derived haematopoietic progenitors to ‘prepare’ an organ for metastasis.^44^ In mice, VEGF mediates the homing of VEGFR-positive haematopoietic precursor cells to form clusters at tumor-specific pre-metastatic sites before the migration of tumor cells.^47^

In ovarian cancer, VEGF overexpression correlates with increased microvascular density, advanced stage of disease, disease recurrence and decreased survival.^48^ VEGF serum levels also correlate with advanced disease, poorly-differentiated ovarian cancer, the presence of ascites and metastases, and poor patient survival.^49,50^ Therefore, it is suggested that VEGF is associated with more aggressive clinical behavior in ovarian cancer, and measurement of VEGF in the serum is a proposed prognostic biomarker in all stages of ovarian carcinoma.^50,51^

#### IL-6

IL-6 is a 20 kDa cytokine that is involved in normal follicle development in the ovary.^52^ However, elevated IL-6 concentrations have been measured in the serum and ascitic fluid of patients with ovarian cancer, and are thought to correlate with volume of disease. Furthermore, high serum levels of IL-6 correlate with shorter survival and are associated with resistance to chemotherapy.^53,54^

Elevated IL-6 may support tumor growth by a number of mechanisms. Although this cytokine does not appear to directly promote tumor cell proliferation, it may influence the migration and invasion of ovarian cancer cells.^52,54-56^ When NOM1 and SKOV ovarian cancer cells were cultured with IL-6, they showed enhanced chemotactic and chemokinetic activity, as well as increased invasiveness.^56^ Invasion of tumor cells is often mediated by the action of extracellular matrix (ECM)-degrading proteinases, such as metalloproteinases. Incubation with IL-6 blocking antibodies resulted in a decrease in the secretion of MMP-9 and MMP-2 by SKOV3 cells in vitro, suggesting that IL-6 acts in an autocrine manner on ovarian carcinoma cells to up-regulate tumorigenic mediators.^52^

Angiogenesis is also enhanced by IL-6. Endothelial cells in both normal ovary and carcinoma specimens have been shown to express functional IL-6 receptors, and thus IL-6 secreted by ovarian carcinoma cells is thought to act on the microvascular endothelium, to trigger a significant angiogenic response. IL-6 enhanced endothelial cell migration in vitro and the degree of angiogenesis in BALB/c mice; however there is mixed evidence for whether this process is dependent on IL-6-mediated VEGF upregulation.^57^

IL-6 acts through its hexameric receptor, which contains the common cytokine receptor signal-transducing subunit, gp130. When IL-6 binds, it triggers signal transduction pathways such as JAK/STAT, Ras/MEK/Erk and PI3K/Akt.^58,59^ These pathways have been implicated in the chemo-resistance of ovarian cancer.^60^

Taken together, IL-6 has a multi-faceted role in the progression of ovarian cancer, and is thus considered to be a potential target for therapy.

#### MIF

Macrophage migration-inhibitory factor (MIF) is a cytokine secreted by granulosa cells that is found in follicular fluids of normal ovarian tissue, but it has critical roles in malignancy, e.g., increasing angiogenesis and tumor cell migration, suppressing p53 activity and activating cyclin D1 and E2F transcription factors. MIF is also involved in tumor immune escape: MIF has been shown to down-regulate natural killer group 2D receptor (NKG2D), an activating receptor on NK cells and a co-stimulatory receptor on CD8+ T cells, leading to inhibition of anti-tumoral NK and CD8+ T cell cytotoxicity.^61^

In ovarian cancer tissue, MIF is strongly overexpressed and significantly higher levels of MIF mRNA have been measured in ovarian carcinoma tissue compared to normal ovarian tissue. Furthermore, elevated MIF protein levels were detected in ascitic fluids from ovarian cancer patients compared to non-malignant ascites and MIF expression levels were significantly higher in patients who had developed ascites compared to those that had not.^61^ Elevated MIF is also detectable in the serum of ovarian carcinoma patients.^62^ Therefore, it has been suggested that MIF may be associated with poor prognosis in ovarian cancer, and may be a promising target for ovarian cancer therapy.

### Immune checkpoint molecules

A number of molecules or receptors that are expressed by immune cells have roles in either suppressing or activating their functions (Fig. 1). By specifically targeting these checkpoint molecules, novel therapies aim to upregulate anti-tumor immune responses, down-regulate pro-tumor functions or depress regulator pathways utilized by tumor cells to subvert anti-tumor responses.

Natural immune responses have been observed in ovarian cancer. Infiltration of immune cells into ovarian tumors was first observed over 30 y ago, and since then an association between T cell infiltration and improved survival in ovarian cancer has been established.^3,63-66^ However, ovarian tumor cells take advantage of a number of mechanisms to evade or suppress this anti-tumor immunity. Targeting checkpoint molecules has shown clinical benefits in other solid tumors such as melanoma and some types of lung carcinomas, prompting studies to test this strategy in ovarian cancer.

#### PD-1 and PD-L1

The programmed cell death 1 (PD-1) co-inhibitory receptor is expressed on activated T cells, and binding of its ligands, PD-L1 and PD-L2, regulates T cell proliferation and immune repsonses.^21^ Ovarian cells are thought to evade anti-tumor immune responses by expressing PD-L1, and thus suppressing T cell activation.^67^ In fact, an inverse correlation has been observed between PD-L1 expression by tumor cells and the number of intra-epithelial CD8+ T lymphocytes in ovarian patient specimens, and high PD-L1 expression by ovarian carcinoma cells is associated with poor prognosis. Therefore, blockade of this receptor/ligand interaction is thought to be a promising therapeutic avenue, which may release the immune blockade to improve anti-tumor immunity. In contrast, there is little evidence to-date to support raised PD-L2 expression in ovarian tumors, with weak or no expression of PD-L2 detected in the same cohort of ovarian cancer patient tissues.^68^

#### CTLA-4

A similar immunoregulatory target, CTLA-4 is a CD28:B7 immunoglobulin superfamily ligand expressed on activated lymphocytes. CD28 signals are known to stimulate T cell activation and survival, whereas the T cell-APC interaction via CTLA-4 inhibits T cell responses, and can lead to T cell arrest and inhibition of effector functions.^69^ Although the activity of CTLA-4 as part of immune evasion in ovarian cancer progression is unclear, therapeutic approaches to prevent its immunosuppressive activity may be beneficial and are being explored.

#### NKG2A

NKG2A is an inhibitory receptor that controls both innate immunity (via NK cells) as well as adaptive immunity (via cytotoxic T cells). Binding of NKG2A to its ligand, HLA-E, which is expressed on tumor cells, results in inhibition of NK and cytotoxic T cells, and thus tumor cells are able to escape destruction by NKG2A+ immune cells. Ovarian cancer is a suitable indication for an NKG2A receptor inhibitor, since HLA-E is upregulated in ∼80% of tumors^70^ and appears to be prognostic.^71^

#### DLL4

Delta-like ligand 4 (DLL4), is an activatory ligand of the Notch signaling pathway, which is an intracellular signaling system that plays a critical role in cell survival, as well as in embryonic development, tissue repair, hematopoiesis, and the maintenance of endothelial and gut epithelial stem cell homeostasis.^72^ Alterations in the Notch pathway can therefore result in the development of malignancies. DLL4 is expressed at sites of angiogenesis, and is the specific ligand of this pathway in endothelial cells. Therefore, DLL4 blockade can result in both ineffective angiogenesis and inhibition of tumor growth.^73^

## Monoclonal antibodies for the treatment of ovarian cancer

Monoclonal antibodies have been developed as vaccines and to target tumor-associated antigens, tumor-promoting molecules and immune checkpoint molecules. Examples of such agents in ovarian cancer are detailed below. Agents that have reached Phase 3 clinical trials are summarized in Table 1. Agents approved for ovarian cancer treatment by the US Food and Drug Administration (FDA), European Medicines Agency (EMA) and UK National Institute for Health and Care Excellence (NICE) are summarized in Table 2.

**Table 1.** Monoclonal Antibodies for treatment of ovarian cancer

Name	Antibody	Phase 3 trial design	Outcome	References
***Monoclonal antibody vaccinations***				
**Abagovomab**	Murine anti-idotypic mAb mimicking CA125	EOC in complete remission following surgery or platinum-based chemotherapy; comparing abagovomab to placebo.	Monthly maintenance injections induced immune response. No improvement in PFS or OS measured.	^75^
^**90**^**Y-muHMFG1**	MUC1-specific murine IgG1 (yttrium-90 labeled)	EOC; ^90^Y-muHMFG1 combined with or compared to standard therapy.	No difference in time to relapse between 2 arms. No improvement in OS with ^90^Y-muHMFG1.	^81^

***Monoclonal antibodies targeting tumor-associated antigens***				
**Catumaxomab**	EpCAM and CD3 bi-specific (trAb)	Combined with paracentesis in recurrent symptomatic malignant ascites resistant to conventional chemotherapy.	Prolonged puncture-free survival and time to next paracentesis, compared to paracentesis alone.	^85^
**Farletuzumab**	FRα-specific humanized IgG1	Platinum-sensitive recurrent OC; comparing carboplatin/taxane treatment alone and with the addition of farletuzumab.	Improved PFS in those with lower CA125 levels.	^97^
		Platinum-resistant OC; comparing paclitaxel alone, or in combination with farletuzumab.	Discontinued when the prespecified criteria for continuation were not met.	NCT00738699
**Trastuzumab**	HER2-specific humanized IgG1	Epithelial OC; comparing triple or doublet combinations of trastuzumab, paclitaxel and carboplatin	Ongoing trial	NCT00011986
**Pertuzumab**	HER2-specific humanized IgG1	Platinum-resistant recurrent OC with low HER3 mRNA expression; comparing pertuzumab combined with topotecan or paclitaxel, or comparing placebo and pertuzumab combined with topotecan, paclitaxel or gemcitabine.	On going trial	NCT01684878

***Monoclonal antibodies targeting tumor-promoting molecules***				
**Bevacizumab**	VEGF-A-specific humanized IgG1	EOC, PPC and fallopian tube cancer; paclitaxel/carboplatin treatment combined with placebo, compared to concurrent bevacizumab, and concurrent plus maintenance bevacizumab.	No improvement in PFS with concurrent bevacizumab compared to chemotherapy, but increased PFS with concurrent plus maintenance bevacizumab. No difference in OS.	^126^
		EOC; carboplatin and paclitaxel alone or in combination with concurrent bevacizumab.	Improved PFS was observed with chemotherapy and bevacizumab combined.	^127^
		Platinum-resistant EOC; paclitaxel, PLD or topotecan alone, or combined with bevacizumab.	Improved PFS with bevacizumab in combination with chemotherapy.	^128^

***Monoclonal antibodies specific for immune targets***				
**Avelumab**	PD-L1-specific human IgG1	Platinum-resistant or refractory OC; comparing avelumab alone, combined with PLD, and PLD alone.	Ongoing trial	NCT02580058

EOC, epithelial ovarian cancer; PFS, progression-free survival; OS, overall survival; trAb, trifunctional antibody; OC, ovarian carcinoma; PPC, primary peritoneal cancer; PLD, pegylated liposomal doxorubicin.

**Table 2.** Monoclonal antibodies approved for ovarian cancer treatment

Name	Antibody	FDA approval	EMA approval	NICE approval
**Catumaxumab**	EpCAM and CD3 bispecific trAb	–	Treatment of EpCAM-positive ascites	Treatment of EpCAM-positive ascites
**Bevacizumab**	VEGF-A-specific humanized IgG1	Treatment of platinum-resistant OC	Treatment of ovarian neoplasms	i. In combination with carboplatin and paclitaxel for the treatment of advanced EOC ii. In combination with carboplatin and gemcitabine for first recurrence of platinum-sensitive EOC, FTC or PPC in patients not previously treated with bevacizumab or other VEGF-targeting agents.

FDA, US Food and Drug Administration, www.fda.gov; EMA, European Medicines Agency, www.ema.europa.eu/ema; NICE, UK National Institute for Health and Care Excellence, nice.org.uk; trAb, trifunctional antibody; OC, ovarian carcinoma; EOC, epithelial ovarian cancer; FTC, fallopian tube cancer; PPC, primary peritoneal cancer.

### Monoclonal antibody vaccinations

#### Abagovomab

Abagovomab is a murine monoclonal anti-idiotypic antibody that mimics parts of CA125. It is designed to act as an active immunogen aimed at breaking immune tolerance to the antigen. A Phase 1 study to evaluate the safety and efficacy of abagovomab in patients with recurrent EOC, individuals with detectable anti-CA125 antibodies demonstrated prolonged survival in contrast to patients who did not show a positive immune response to the vaccination. In a Phase 1b/2 trial, 68% of patients developed a specific anti-idiotypic antibody response, and cytotoxicity against CA125-positive cancer cells was detected in 27% of patients. Moreover a correlation between the detection of anti-anti-idiotypic antibodies (Ab3) and survival time was observed.^74^

Long-term efficacy of abagovomab was evaluated in a Phase 3 study (NCT00418574) in ovarian cancer patients who were in complete clinical remission after primary surgery and platinum-based chemotherapy. Monthly maintenance injections of abagovomab were considered to be safe and induced a robust immune response; however, treatment did not prolong PFS or OS.^75^

#### Oregovomab

The murine, anti-CA125 IgG1 antibody, oregovomab, readily binds antigen-presenting cells (APCs) when complexed with CA125, leading to the production of anti-CA125 antibodies, and subsequent B and T cell immune responses.^76^ Clinical benefit of this antibody was first observed when used as a technetium-99m-labeled radiodiagnostic, and it has been evaluated in a number of Phase 2 and 3 trials.

In patients with recurrent ovarian carcinoma, oregovomab was well-tolerated, and both human anti-mouse antibody (HAMA) and anti-idotype antibody immune responses were measured.^77,78^ Increases in T cell immune responses to CA125, autologous tumor and oregovomab were measured in 39%, 63% and 50%, respectively, of patients undergoing continued oregovomab and chemotherapy treatment.^77^ Patients who mounted immune responses to CA125 or autologous tumor demonstrated significantly improved survival compared to those who did not. However, oregovomab monotherapy did not induce such significant clinical outcomes in Phase 2 studies in patients following initial first-line surgical and chemotherapy treatment. A subpopulation of patients with optimal surgical outcomes appeared to demonstrate a possible clinical response to oregovomab; however, these results were not repeated in a larger multi-center Phase 3 placebo-controlled trial.^79^ This may be due to the significant reduction in circulating CA125 in these post-surgery patients, resulting in a relative depression of immune response augmented by oregovomab. Given that the most promising outcomes were observed in patients undergoing concomitant chemotherapy,^77^ current clinical trials aim to evaluate oregovomab in combination with carboplatin and paclitaxel (NCT01616303). Oregovomab was granted US and EU orphan designations for treatment of ovarian cancer.

#### HMFG1

HMFG1 is a murine monoclonal IgG1 antibody that recognizes a specific epitope (PDTR) on human MUC1. The first Phase 1 clinical study in patients with EOC, who had undergone at least one regimen of platinum-based therapy, demonstrated the safety and tolerability of repeated intradermal injections of HMFG1. Furthermore, anti-idiotypic antibodies were detected in all patients after 3 vaccinations.^80^ A large Phase 3 trial in patients with EOC who had undergone a surgically defined complete remission of the disease, was then carried out to compare yttrium-90 labeled murine HMFG1 (^90^Y-muHMFG1), plus standard therapy, with standard therapy alone. However, no difference in time to relapse was detected between the 2 arms of the study, and a single injection of ^90^Y-muHMFG1 did not improve OS.^81^

### Monoclonal antibodies targeting tumor-associated antigens

#### PankoMab-GEX

PankoMab-GEX is a glycoengineered humanized IgG1 mAb specifically targeting the PDTRP epitope of MUC1. The optimized glycosylation of PankoMab-GEX leads to enhanced antibody-dependent cell-mediated cytotoxicity and phagocytosis (ADCC/ADCP).^82^ In a Phase 1 study (NCT01222624) in patients with advanced MUC1-expressing solid tumors, PankoMab-GEX was well tolerated; no maximum tolerated dose (MTD) was reached and AEs were mainly mild to moderate infusion-related reactions. Clinical benefit was observed in 47% of patients, including 1 complete response (CR) in a patient with ovarian carcinoma, and confirmed stable disease (SD) in 19 patients.^82^ There is now an ongoing placebo-controlled Phase 2 study (NCT01899599) of PankoMab-GEX as maintenance therapy in advanced ovarian, fallopian tube or primary peritoneal cancer.

#### Catumaxomab

Catumaxomab is a bispecific trifunctional antibody (trAb) specific to both EpCAM and CD3, which is expressed on T cells. The unique hybrid Fc portion of the antibody is composed of mouse IgG2a and rat IgG2b and selectively binds type I, IIa and III human Fcγ receptors expressed on accessory immune cells such as macrophages, NK cells and dendritic cells,^83^ leading to ADCC and ADCP, and activation of T-cell mediated tumor cell cytotoxicity.^84^

In a Phase 1/2 study of catumaxomab in ovarian cancer patients with ascites containing EpCAM-positive tumor cells, significant ascites reduction was observed and minimal adverse events (AEs) occurred.^83^ A subsequent Phase 2/3 trial comparing paracentesis plus catumaxomab with paracentesis alone showed that addition of catumaxomab significantly prolonged puncture-free survival and time to next paracentesis. Furthermore, patients given catumaxomab suffered fewer symptoms from ascites and had a positive trend in OS.^85^ Catumaxomab has EMA and NICE approval for use in EpCAM-positive ascites treatment (see Table 2).

#### chMOv18 IgG

chMOv18 IgG is a human chimeric IgG1 antibody specific for FRα, cloned from a murine MOv18 IgG antibody that was generated by immunization of mice with a surgical specimen of ovarian cancer.^86^ Following studies showing effective tumor uptake and anti-tumor efficacy of radiolabeled murine MOv18 IgG,^87^ radiolabeled chMOv18 IgG was shown to localize well into the tumor tissue of ovarian cancer patients.^88^ Results of a Phase 1 trial, in which chMOv18 IgG given intravenously (i.v.) at increasing doses, indicated that the antibody was safe with no significant hematological or biochemical changes.^89^

The route of administration for radiolabeled chMOv18 IgG was evaluated in 2 studies comparing i.v administration with locoregional delivery via intraperitoneal (i.p.) administration.^90,91^ Radiolabeled chMOv18 IgG given either i.p or i.v. was considered to give no normal organ toxicity, with no AEs observed. One study suggested that that better accumulation of antibody occurred after locoregional i.p administration, compared to i.v.,^90^ but the other study did not measure any advantage. Use of the i.p route of administration was recommended with respect to bone marrow toxicity, as the area under the blood activity vs. time curve (AUC) was significantly lower for the i.p. route.^91^

#### Farletuzumab

Farletuzumab (MORab003) is a humanized IgG1 antibody specific for FRα. Following in vitro studies showing a number of mechanisms of anti-tumor activity,^92,93^ a Phase 1 study, in patients with platinum-resistant EOC was carried out to establish the MTD.^94^ The safety of farletuzumab in combination with carboplatin and pegylated liposomal doxorubicin in patients with platinum-sensitive EOC was then demonstrated in a Phase 1b study.^95^

Promising overall response rates were observed in a Phase 2 study in patients with platinum-sensitive ovarian cancer given farletuzumab/carboplatin/taxane combination therapy, followed by farletuzumab maintenance therapy. Furthermore, farletuzumab did not lead to additive toxicity when combined with chemotherapy.^96^ Subsequently, a Phase 3 trial was carried out in platinum-sensitive patients with recurrent ovarian cancer compared the carboplatin/taxane treatment alone with the addition of farletuzumab. Here, improved PFS was observed in patients with lower CA125.^97^ A second Phase 3 study in patients with platinum-resistant ovarian cancer compared paclitaxel alone, or in combination with farletuzumab (NCT00738699); however this trial was discontinued when the pre-specified criteria for continuation were not met.

The manufacturer of farletuzumab has since announced the development of a diagnostic assay to identify patients with high FRα expression, suggesting that farletuzumab trials in the future may stratify patients according to tumor target expression.^98^ The suggestion that farletuzumab may be most beneficial in patients with low CA125 serum levels is being addressed in a Phase 2 study comparing farletuzumab, combined with carboplatin and paclitaxel, or with carboplatin and pegylated liposomal doxorubicin (NCT02289950).

#### Amatuximab

Amatuximab (MORAb-009) is a high-affinity chimeric IgG1 mAb targeting mesothelin. Following promising preclinical studies,^99^ a Phase 1 study of amatuximab in 24 patients with treatment-refractory, mesothelin-expressing solid tumors was initiated. In this study, amatuximab was well tolerated, an MTD of 200 mg/ml was determined and 11 patients experienced SD, with no observed objective responses. The most common AE was drug hypersensitivity consisting of infusion reactions/cytokine release syndrome and allergic reactions.^100^ To date, no Phase 2 trials in ovarian cancer have been planned for this antibody.

#### Cetuximab

Cetuximab is a chimeric IgG1 antibody specific for the extracellular domain of EGFR (i.e., HER1) that is approved for use in colorectal and head and neck cancers. Upon binding, the antibody prevents signaling via EGFR, and accelerates internalization of the receptor.^101^ Cetuximab demonstrated anti-tumor activity and augmentation of the efficacy of chemotherapies such as cisplatin, doxorubicin, paclitaxel, and topotecan both in vitro studies and in human xenograft animal models.^102^ In a Phase 2 trial of cetuximab as a single agent in patients with persistent/recurrent ovarian cancer, minimal clinical benefit was observed, with 9 patients achieving SD and only 1 patient demonstrating a partial response (PR).^103^ Furthermore, in a Phase 2 study of cetuximab, combined with paclitaxel and carboplatin, in patients with stage III/IV ovarian cancer, this combination therapy was adequately tolerated, but did not demonstrate prolonged PFS compared to historical data.^104^ More promising results were observed in another Phase 2 study, where cetuximab was combined with carboplatin in platinum-sensitive ovarian cancer patients. Here 9 of 29 patients had an objective response and 8 patients had SD. As previously observed, cetuximab therapy was associated with an acneiform rash in the majority of patients, and occasional serious hypersensitivity reactions were observed.^105^

Although meaningful efficacy data is lacking for the use of cetuximab in ovarian cancer, future developments may include the use of pharmacogenetic data (such as K-RAS mutation status) to select appropriate patients for treatment, as has been carried out for colorectal cancer, or cetuximab may be used in combination VEGF inhibitors such as bevacizumab.^103,104^

#### Panitumumab

Panitumumab is a human recombinant IgG2 mAb approved for use in metastatic colorectal cancer.^106^ It binds to the extracellular domain of EGFR/HER1, preventing its activation.^107^ In a Phase 2 study, platinum-resistant, K-RAS wild-type, EOC patients were given panitumumab in combination with pegylated liposomal doxorubicin. Overall response as determined by CA125 levels was observed in 18.6% of the intent-to-treat population. Using RECIST evaluation, 9.3% of patients had a PR, 18.6% had SD and 39.5% progressed on treatment. The most common treatment-related grade 3 toxicities were skin toxicity, fatigue and vomiting, and skin toxicities were the major reason for dose reductions. Overall, although a moderate response rate was observed, the occurrence of skin toxicities was considered to have a substantial effect on the quality-of-life of patients.^108^ The K-RAS mutation status of patients may be a useful molecular marker for selection of patients to treat with panitumumab.

Panitumumab in combination with everolimus and bevacizumab was also evaluated in a Phase 1 study in patients with advanced solid tumors.^107^ Three of the 32 subjects had ovarian cancer, and these individuals showed prolonged disease control ranging from 11 to >40 months. The recommended combination dose regimen showed acceptable safety and tolerability and moderate clinical activity, particularly in gynecological cancers.^107^ More recently, a Phase 2 trial of panitumumab combined with gemcitabine in patients with relapsed ovarian cancer was terminated because newer technologies are available and the study had poor accrual (NCT01296035).

#### Matuzumab

Matuzumab (EMD72000) is a humanized IgG1 mAb that binds to EGFR/HER1 at the ligand-binding region. It is thought that matuzumab acts by inhibiting EGFR binding and signaling, and by triggering ADCC against EGFR-expressing tumor cells.^109^ Results of a Phase 1 studies in colon cancer patients showed matuzumab was well tolerated, with the predominant toxicities being those of the skin.^110^ A Phase 2 trial in patients with recurrent, EGFR-positive ovarian cancer demonstrated that the therapy was reasonably well tolerated, although no formal responses of matuzumab efficacy were observed.^109^

#### Trastuzumab

Trastuzumab is a humanized IgG1 antibody that binds to the extracellular domain IV of HER2, preventing activation of the intracellular tyrosine kinase, and thus inhibiting proliferation of cells overexpressing HER2.^111^ It is approved for breast cancer and adenocarcinoma of the stomach. On the basis of the observed safety of trastuzumab in breast cancer, a single-arm Phase 2 trial of trastuzumab was carried out in persistent or recurrent ovarian cancer patients selected on the basis of high levels of HER2 tumor expression. Only a 7.3% response rate and 39% disease stabilization rate was observed in these patients. However, several patients that responded or showed SD continued to receive trastuzumab therapy for more than 1 year, demonstrating the overall tolerability of treatment; reported drug related toxicities tended to be mild and not dose-limiting. Furthermore, data from this study suggested that there was no correlation between the level of HER2 expression and response to trastuzumab treatment.^111^ Another Phase 2 study of trastuzumab combined with a carboplatin-paclitaxel regimen was also initiated in patients with ovarian cancer that had previously relapsed within 6 months of carboplatin-paclitaxel treatment and had high levels of tumor HER2 expression. This trial has since been terminated with no reason reported (NCT00189579).

A Phase 3 trial (NCT00011986) of trastuzumab in triplet or doublet treatment combinations with paclitaxel and carboplatin in patients with EOC has been reported as complete, although results have not yet been published. A number of other Phase 1 studies evaluating trastuzumab in combined with interleukin-12 have also been carried out, with results yet to be reported (NCT00028535 and NCT00004074).

#### Pertuzumab

Pertuzumab (rhuMAb 2C4) is an IgG1 humanized mAb that binds to the extracellular domain II of HER2, preventing hetero-dimerization with HER1 or HER3. It is approved for use in breast cancer treatment,^112^ but has shown efficacy against ovarian cancer cells in vitro.^113-116^ In the Phase 1 study in patients with advanced solid tumors, pertuzumab was well tolerated, and 2 of the 21 patients, including one with ovarian cancer, achieved a PR.^117^ In a Phase 2 trial, pertuzumab alone was evaluated in platinum-resistant ovarian cancer. Overall, 14.5% of participants were considered to have achieved any clinical benefit.^118^ In a larger Phase 2 trial, pertuzumab combined with gemcitabine was compared to gemcitabine plus placebo in platinum-resistant patients. The objective response rate was 13.8% in patients receiving the combination, compared to 4.6% in those receiving gemcitabine and placebo. This increased benefit of pertuzumab was shown to occur more in patients with lower HER3 mRNA expression, whereas patients with high HER3 tumor expression were less likely to benefit from pertuzumab addition to treatment. In this study, an increased incidence of grade 3 and 4 neutropenia, thrombocytopenia, back pain and diarrhea were observed in the combination arm.^115^ The efficacy of pertuzumab was also evaluated in a Phase 2 trial in combination with carboplatin and paclitaxel or gemcitabine in platinum-sensitive patients; however no significant differences in PFS or other secondary endpoints were observed between the cohorts.^119^

Although HER2 expression by ovarian cancer and breast cancer cells is comparable, efficacy of pertuzumab in ovarian cancer is not equivalent to that seen in treating breast cancer.^112^ This may be dependent on HER3 expression^115^ or HER2 activation.^114^ Therefore, future pertuzumab studies are likely to include stratification of patients using such biomarkers. There is an ongoing Phase 3 study combining pertuzumab and standard chemotherapy in platinum-resistant ovarian cancers with low HER3 mRNA expression (NCT01684878), and another Phase 2 trial evaluating HER2 activation on pertuzumab efficacy in advanced ovarian cancer has just been completed, with results yet to be reported (NCT00058552).

#### Seribantumab

Seribantumab (MM-121, SAR256212) is a human HER3-targeting IgG2 mAb that prevents the dimerization of HER2 and HER3 and inactivates downstream signaling.^120^ This antibody has recently been evaluated in combination with paclitaxel in a randomized Phase 2 study of platinum resistant advanced ovarian and fallopian tube cancer (NCT01447706). Most reported AEs were mild to moderate; however PFS was not prolonged by combination of seribantumab with paclitaxel.^121^

#### Olaratumab

Olaratumab (IMC-3G3), a human IgG1 mAb that selectively binds PDGFRα, prevents ligand binding^122^ and significantly enhances the efficacy of chemotherapy against ovarian tumor cells in vitro and in vivo.^123^ In a Phase 1 study in patients with solid tumors olaratumab was well tolerated and no DLTS were observed so the MTD was not identified. No CR or PR was measured, but SD was measured in 12 of the 19 evaluable patients and RP2Ds of 16 mg/kg weekly and 20 mg/kg biweekly were determined. ^122^ A Phase 2 study (NCT00913835) evaluating the efficacy of PLD, with or without olaratumab, in platinum-refractory or resistant advanced ovarian cancer has been conducted and results are awaited.

### Monoclonal antibodies targeting tumor-promoting molecules

#### Bevacizumab

Bevacizumab, a recombinant humanized monoclonal IgG1 antibody specific to all biologically active forms of VEGF-A, has demonstrated anti-angiogenic activity and inhibition of ovarian cancer progression in in vitro studies and animal models.^124^ In a Phase 2 study in patients with recurrent EOC and primary peritoneal cancer (PPC), 3% and 18% of patients showed CR and PR, respectively, and 40% of patients showed at least 6 months PFS following treatment.^125^

A number of Phase 3 trials of bevacizumab have been completed. In patients with EOC, PPC and fallopian tube cancer, paclitaxel/carboplatin treatment combined with placebo was compared to concurrent bevacizumab, and concurrent plus maintenance bevacizumab. Although concurrent bevacizumab treatment showed no improvement in PFS compared to the chemotherapy arm, patients who also received concurrent plus maintenance bevacizumab had an increased PFS of 3.8 months. However, no difference in OS was observed. Bevacizumab treatment was well tolerated, with the major AEs attributed to chemotherapy.^126^ In another Phase 3 trial, patients with EOC were treated with carboplatin and paclitaxel alone or in combination with concurrent bevacizumab. Here, improved PFS was observed in those receiving chemotherapy combined with bevacizumab.^127^ The efficacy of bevacizumab in combination with chemotherapy has also been studied in a Phase 3 trial in patients with platinum-resistant ovarian cancer. Patients were treated with paclitaxel, pegylated liposomal doxorubicin (PLD) or topotecan alone, or combined with bevacizumab. Those receiving bevacizumab in combination with chemotherapy showed significantly improved PFS.^128^ The observed improvements in these studies may not only be due to the anti-angiogenic effect of bevacizumab, but may also be attributed to bevacizumab–related normalization of tumor vasculature, which may lead to enhanced delivery of chemotherapeutic drugs. Bevacizumab is approved for ovarian cancer treatment by the FDA, EMA and NICE (see Table 2).

#### Volociximab

Volociximab is a first-in-class chimeric IgG4 mAb that targets human α5β1 integrin with high affinity. Preclinical studies of this antibody, which showed inhibition of VEGF- and bFGF-induced neovascularization, associated reduction in tumor burden, ascites and number of metastases, and increased survival in animal models of ovarian cancer,^129^ were followed by a number of clinical trials. In a Phase 1 study, no DLTs were observed and dose reduction was not required. Of the 20 patients evaluable for efficacy, 1 patient with renal cell carcinoma had a minor response and 5 others had durable SD. No CRs or PRs were observed.^130^ Subsequently, a multicenter Phase 2 single-arm, 2-stage study in platinum-resistant advanced EOC or primary peritoneal cancer was carried out. Of the 14 patients evaluable for efficacy, 1 had SD at 8 weeks, but the remaining 13 patients all demonstrated PD. This lack of efficacy may have been caused by the chemotherapy-resistant study population. In addition, 75% of patients experienced AEs, primarily headache and fatigue.^131^ In a second Phase 2 study, patients with EOC were stratified by platinum sensitivity and treated with PLD or volociximab alone, or the combination.^132^ AEs and response rates were similar across treatment groups.^132^

#### Siltuximab

Siltuximab (CNTO 328, cCLB8) is a chimeric mAb that binds to IL-6, neutralizing its effects in a number of human malignancies including ovarian cancer. Following in vitro analyses showing that the intensity of IL-6 immunohistochemistry (IHC) staining of ovarian cancer tissues was associated with poor patient prognosis, and that siltuximab had tumor growth efficacy, a single arm, Phase 2 study of siltuximab in patients with recurrent, platinum-resistant ovarian cancer was conducted. Although the response rate to siltuximab, the primary endpoint of the study, was modest, prolonged periods of disease stabilization were observed. Median OS was similar to that of large Phase 2 trials of conventional chemotherapy in platinum-resistant ovarian cancer.^133^ Plasma cytokine/chemokine levels were also almost unchanged following 3 siltuximab infusions; however, patients who received at least 6 months of infusions showed lower levels of IL-6-regulated chemokines, CCL2 and CXCL12, and the angiogenic molecule VEGF. Efficacy of siltuximab was also evaluated in a Phase 1/2 study in patients with advanced solid tumors, including EOC. This study was successful in demonstrating that siltuximab monotherapy is well tolerated, but it did not demonstrate clinical activity.^134^ No additional studies have been initiated in ovarian cancer.

#### Imalumab

Imalumab (Bax69) is a human IgG1 mAb that targets oxidized MIF (oxMIF) to reduce tumorigenesis. Interim reports of a Phase 1 study in solid tumors (NCT01765790), report that imalumab was well tolerated and no MTD had been reached. SD was measured in 7 heavily pretreated patients and a recommended Phase 2 dose (RP2D) of weekly 10 mg/kg i.v. was determined.^135^ A Phase 1/2a study of imalumab is now recruiting patients with ovarian carcinoma and recurrent malignant ascites (NCT02540356). In this study, imalumab monotherapy will be administered i.p. (study single-route arm) and compared to i.v. followed by i.p. infusions (double-route arm).

### Monoclonal antibodies specific for immune targets

#### Nivolumab

Nivolumab is a human anti-PD-1 IgG4 mAb. A Phase 1 study in patients with advanced tumors demonstrated clinical responses to nivolumab. The greatest rate of response was observed in melanoma patients; of the 17 ovarian cancer patients treated, 6% had a PR and 18% had SD.^136^ In a Phase 2 trial in platinum-resistant ovarian cancer the overall response rate to nivolumab was 15%, and the disease control rate was 45%. The median OS for the cohort was 20 months.^137^ Strategies for patient stratification are still unclear as no significant correlation between clinical response and patient tissue PD-L1 expression was identified.^137^

#### Pembrolizumab

Recently, interim results were presented from a Phase 1b study of pembrolizumab, a humanized anti-PD-1 IgG4 mAb. Of the 26 patients with PD-L1-expressing advanced ovarian cancer who were treated with pembrolizumab, 1 patient had a CR, 2 achieved PR, and 6 had SD, resulting in a disease control rate of 34.6%.^138^

#### BMS-936559

A human anti-PD-L1 IgG4 mAb, BMS-936559, has been evaluated in a Phase 1 study that included patients with ovarian cancer. In this study, 6% of patients receiving the MTD of 10 mg/kg achieved PR and 18% had SD lasting more than 24 weeks.^139^

#### Avelumab

A human IgG1 mAb targeting PD-L1, avelumab (also known as MSB0010718C) is unlike other PD-L1 targeting mAbs as it is able to mediate ADCC of tumor cells (due to its functional IgG1 Fc region). In a Phase 1 study in patients with refractory cancers, including ovarian carcinoma, significant inhibition of the PD-L1 receptor on peripheral blood lymphocytes (PBLs) was measured following avelumab treatment. To-date, the data from this study are still being collected. However, preliminary results from an ongoing Phase 1b study in patients with recurrent or refractory ovarian cancer (NCT01772004) have shown that 17.4% of patients had a PR and 47.8% had SD.^140^ A Phase 3 study to evaluate avelumab alone, compared to avelumab combined with PLD, and PLD alone, is now recruiting patients with platinum-resistant or refractory ovarian cancer (NCT02580058).

#### Ipilimumab

Ipilimumab, a human IgG1 targeting CTLA-4, was approved by the FDA in 2011 for treatment of metastatic or unresectable melanoma. The mAb was well tolerated in a Phase 1/2 trial in patients with stage IV ovarian carcinoma, who had previously either received chemotherapy or granulocyte-macrophage colony-stimulating factor modified irradiated autologous tumor cells (GVAX).^141,142^ One ovarian cancer patient had a significant decrease in serum CA125 levels and tumor regression, and an additional 5 patients showed stabilization of CA125 levels.^142^ Furthermore, the therapeutic effects of ipilimumab were associated with anti-NY-ESO-1 antibody responses. Tumor regression also correlated with the CD8+/Treg ratio, suggesting that other Treg-depleting therapies may be combined with ipilimumab for a synergistic effect. Another Phase 2 study (NCT01611558) of ipilimumab monotherapy in patients with platinum-sensitive ovarian cancer is ongoing.

#### Tremelimumab

Tremelimumab is a human IgG2 mAb that targets CTLA-4. An ongoing Phase 1/2 trial (NCT02571725) is evaluating the combination of tremelimumab with the PARP-inhibitor olaparib in patients with BRCA-deficient recurrent ovarian cancer. Phase 1 studies (NCT02261220, NCT01975831) of tremelimumab combined a PD-1 inhibitor, MEDI4736, in patients with solid tumors including ovarian cancer are also now recruiting.^143^

#### Monalizumab

Monalizumab (IPH2201) is a humanized IgG4 mAb that blocks the binding of NKG2A to HLA-E, allowing activation of NK and cytotoxic T cell responses. IPH2201 may also enhance the cytotoxic potential of other therapeutic antibodies. In a Phase 1 dose-escalation study, monalizumab was safe and well-tolerated.^144^ It is currently under evaluation in a Phase 1/2 trial (NCT02459301) of platinum-resistant or sensitive patients with high grade ovarian cancer.

#### Demcizumab

Demcizumab (OMP-21M18) is a humanized IgG2 mAb targeting DLL4. The Phase 1 study of demcizumab demonstrated initial single-agent activity, including early evidence of durable anti-tumor responses. This study included one patient with an ovarian granulosa cell carcinoma who demonstrated SD as best response, and remained on treatment for 17 months.^145^ Overall, therapy was generally well tolerated; however, the prolonged exposure to demcizumab, as with the patient with ovarian granulosa cell carcinoma, resulted in delayed cardiac toxicity and tumor-associated bleeding consistent with dysfunctional angiogenesis, necessitating termination of the study.^145^ A Phase 1b/2 study (NCT01952249) of demcizumab, plus paclitaxel, is now recruiting patients with platinum-resistant ovarian, primary peritoneal or fallopian tube cancer.

### Antibody-drug conjugates

An antibody-drug conjugate (ADCs) directs the potent activity of a small molecule cytotoxic agent to the appropriate tumor cells by covalently attaching the agent to a tumor antigen-specific mAb. Most cytotoxic agents used as payloads in ADCs are potent microtubule inhibitors or DNA damaging agents, and do not have a therapeutic index when administered as free drugs.^146^

#### DMUC4064A

DMUC4064A is an ADC composed of a CA125-targeting humanized IgG1 conjugated to an anti-mitotic agent, MMAE. A Phase 1 study (NCT02146313) of DMUC4064A in patients with platinum-resistant ovarian cancer or unresectable pancreatic cancer is ongoing.

#### IMGN853

IMGN853 (mirvetuximab soravtansine) is a FRα-targeting ADC composed of a humanized FRα-binding antibody attached to a highly potent maytansinoid, DM4, that induces cell-cycle arrest and cell death by targeting microtubules.^147^ A Phase 1 study in patients with EOC or other FRα-positive solid tumors, demonstrated encouraging clinical activity in heavily pretreated patients, with a manageable AE profile.^148^ Clinical benefit (PR or CR, CA125 response, SD ≥ 6 cycles) was observed in 11/44 patients.^148^ In another trial, the RP2D showed clinical benefit in 5/10 evaluable patients with platinum-resistant EOC. A Phase 1b study of IMGN853 administered with bevacizumab, carboplatin, or PLD, in patients with FRα-positive advanced EOC, primary peritoneal cancer, fallopian tube cancer or endometrial cancer is open to recruitment (NCT02606305). Furthermore, a Phase 2 study (NCT02631876) is recruiting patients with FRα-positive advanced EOC, primary peritoneal cancer or fallopian tube cancer to compare IMGN853 with the investigator's choice of chemotherapy.

#### DNIB0600A

DNIB0600A (lifastuzumab vedotin) is a humanized IgG1 anti-NaPi2b mAb conjugated to an anti-mitotic agent, MMAE, that has shown anti-proliferative activity in xenograft models.^149^ In a Phase 1 study of DNIB0600A in patients with non-small cell lung cancer or platinum-resistant ovarian cancer, archival tumor NaPi2b expression was evaluated by IHC.^150^ At the recommended Phase 2 dose, 41% of IHC 2/3+ patients with ovarian cancer had confirmed PRs. No patient with an IHC score of 0 showed a clinical response.^151^ DNIB0600A in combination with carboplatin, and with or without bevacizumab is now being evaluated in a Phase 1b study (NCT01995188) in patients with platinum-sensitive ovarian cancer. A Phase 2 randomized trial (NCT01991210) of DNIB0600A, in comparison to PLD, in patients with platinum-resistant ovarian cancer is also ongoing.

#### PF-06647263

PF-06647263 is a first-in-class, humanized IgG2 mAb (huE22) specific for EFNA4, and linked to calicheamicin, a DNA-damaging cytotoxic agent.^43^ Initial results from a Phase 1 study in patients with advanced solid tumors unselected for EFNA-4 expression show a DLT in only 1 patient, and 2 patients (1 with ovarian cancer and one with triple negative breast cancer) have experienced a PR.^151^

#### SC-003

SC-003 is an ADC composed of a proprietary TAA-specific mAb conjugated to an undisclosed cytotoxic agent with unpublished mechanism of action.^152^ A Phase 1 study (NCT02539719) of SC-003 is now recruiting patients with platinum-resistant, refractory ovarian cancer.

### New directions for ovarian cancer immunotherapy

#### IMAB027

A proportion of advanced stage ovarian cancers express high levels of Claudin 6 (CLDN6), a carcino-embryonic transmembrane protein that is not found on normal adult human tissue. IMAB027 is a mAb that binds to CLDN6. Pre-clinically, this antibody was shown to inhibit tumor growth and to kill cancer cells by ADCC and complement-dependent cytotoxicity (CDC). A Phase 1/2 trial (NCT02054351) of IMAB027 in patients with recurrent advanced ovarian cancer is currently underway. Early data suggests that IMAB027 was well tolerated, with no DLTs observed, and the MTD was not reached.^153^

#### MOv18 IgE

MOv18 IgE is a FRα-specific chimeric IgE antibody engineered from MOv18 IgG1.^154^ Following trial results of farletuzumab and chMOv18 IgG1, where FRα was shown to be a promising target and chMOv18 IgG1 was well tolerated (described above), MOv18 IgE was developed to investigate the hypothesis that antibodies engineered with Fc regions of the IgE class may offer advantages over their IgG counterparts.^155^ MOv18 IgE afforded superior protection compared with its IgG1 counterpart in a number of in vivo models of ovarian cancer,^156,157^ and is now undergoing evaluation in a Phase 1 clinical study (NCT02546921).

## Conclusion and future perspectives in antibody design

Improved understanding of the contribution of tumor-associated molecules to ovarian cancer growth and spread has helped identify known and novel targets for therapy. Alongside novel emerging targets involved in immune regulation, these insights are now yielding a range of different treatment approaches involving monoclonal antibodies.

One area likely to receive increasing attention may be antibody engineering strategies aimed at enhancing Fc-mediated antibody mechanisms of action and bioavailability.^158^ These may include: 1) engineering of antibodies or antibody fragments with specificity for more than one target (e.g., bispecific antibodies), with the aim of simultaneously recruiting immune effector cells to the tumor target; 2) engineering of differentially glycosylated Fc regions in order to enhance antibody bioavailability and augment the capacity of antibodies to trigger ADCC; or 3) mutational engineering of antibody Fc regions with the aim of improving both ADCC and CDC. Beyond antibody engineering techniques, attempts at augmenting Fc-mediated responses have been made, by strategies to increase the activation, expansion and recruitment of effector cells such as by co-administering the antibody with recombinant cytokines.

All currently approved mAbs are of the IgG isotype^159^ due to favorable pharmacokinetic properties and the pioneering experiments by Neuberger and colleagues, which demonstrated that IgG, and in particular IgG1, was more effective than other antibody isotypes in activating complement and promoting ADCC and CDC against tumor cells in vitro. Different IgG antibody subclasses, however, have been employed therapeutically, such as IgG1, IgG2 and IgG4, depending on the desired role of the Fc-mediated mechanisms of action. The IgG1 Fc region generally has the highest affinity of all of the IgG antibody subclasses for Fcγ receptors (FcγRs) on immune effector cells, leading to more potent effector functions.^160^ On the other hand, IgG4 has weaker affinity for FcγRs, which translates into weaker or ineffective effector functions. Therefore, IgG4 mAbs are used therapeutically when Fc-mediated effector functions are not desirable.^136^ Furthermore, there has been some exploration of the use of mAbs of alternate Ig classes such as IgA and IgE in cancer immunotherapy, with the aim of enhancing Fc-mediated effector functions at anatomic locations where tumors may be located, and through the engagement of cognate FcRs on immune effector cells such as FcαRI, FcϵRI, and CD23.^154-157^

Another area likely to expand will be the identification of effective combinatory treatments. Single-agent immunotherapies have produced promising clinical responses in ovarian carcinoma and other tumors. However, to achieve the maximal anti-tumor immune response, it is likely that combination therapeutic strategies will be required. For many tumors, several ‘tumor escape mechanisms’ acting in parallel allow tumors to evade detection by the immune system.^161^ The concept behind a combinational immunotherapy strategy is to target tumor escape mechanisms at different stages, thereby creating the possibility of synergistic effects between agents and potentially benefiting a greater proportion of patients.

An example of a combination antibody immunotherapy strategy in ovarian cancer involves the combination of multiple immune checkpoint blockades. In preclinical studies, almost half of the tumor-infiltrating lymphocytes in an in vivo model of ovarian carcinoma were positive for both CTLA-4 and PD-1, and demonstrated an attenuated capacity to proliferate and produce cytokines.^162^ Treating these mice with both anti-PD-1 and anti-CTLA-4 antibodies resulted in reversal of the tumor-infiltrating lymphocyte dysfunction, and induced tumor regression in 50% of the mice compared to 25% with either agent as a monotherapy.^162^ A Phase 1/2 trial evaluating nivolumab and the IDO inhibitor INCB24360 in patients with ovarian cancer is currently underway (NCT02327078). Since the IDO pathway leads to the expansion of Tregs, combining an agent that enhances effector T cell function with an agent that restricts immunosuppressive elements such as myeloid-derived suppressor cell (MDSC), Tregs, and macrophage subtypes in the tumor microenvironment may be more efficacious than either agent as monotherapy. In another study, a dendritic cell-based autologous whole-tumor lysate vaccine combined with bevacizumab was evaluated in patients with ovarian cancer. Four of 6 patients demonstrated a clinical benefit, in addition to an increase in tumor-reactive T cells (as measured by IFN-γ secretion).^163^

Although it is likely that the interest in novel combination immunotherapy studies in ovarian cancer will increase, simultaneous application of multiple immunotherapies requires careful consideration of: 1) the potential for overlapping toxicities; 2) the need to optimize the timing of administration of each agent (sequential vs. concurrent); and 3) the critical issue of patient stratification, e.g., which patients need combination strategies and what combinations are best in any given patient. A proportion of patients treated with monotherapy demonstrate prolonged responses, suggesting that some patients may not need combination immunotherapy. There is an urgent need to develop biomarkers to identify such patients to preselect them for each monotherapy in order to avoid the toxicities associated with combination immunotherapy. If all of these considerations can be managed, it is likely that combination immunotherapy, and our improved understanding of mechanisms of immune modulation in ovarian carcinoma, may dramatically improve the clinical outcomes of ovarian cancer patients.
